# Accuracy of Provisional Reports by Radiology Residents on Call

**DOI:** 10.7759/cureus.118704

**Published:** 2026-10-28

**Authors:** Mariam Dean, Hammad Qadi, Ali Rana, Kar Yan-Yip, Anmol Gangi Burton

**Affiliations:** 1 Department of Trauma and Orthopaedics, Wrightington, Wigan and Leigh NHS Foundation Trust, Manchester, GBR; 2 Department of Radiology, Nottingham University Hospitals NHS Trust, Nottingham, GBR; 3 Department of Radiology, Doncaster and Bassetlaw Teaching Hospitals NHS Foundation Trust, Doncaster, GBR; 4 Department of Radiology, Royal Stoke University Hospital NHS Trust, Stoke, GBR

**Keywords:** ct, diagnostic error, discrepancy rate, mri, out-of-hours reporting, patient safety, radiology residents, workload

## Abstract

Background

Out-of-hours (OOH) CT and MRI examinations at our institution are provisionally reported by on-call radiology residents and used to guide overnight patient management. We aimed to quantify the overnight discrepancy rate between provisional resident reports and subsequent consultant reports, and explore associations between discrepancies and resident grade, study type, and reporting time.

Methods

This registered service evaluation retrospectively reviewed all consecutive CT and MRI examinations provisionally reported overnight by radiology residents between 13 June 2022 and 3 July 2022. Provisional reports were compared with the final consultant reports. Discrepancies were categorised as minor, significant, or major using predefined criteria by an ST4 radiology resident.

Results

In total, 993 imaging examinations were included. The overall discrepancy rate was 144 (14.5%). Major and significant discrepancies comprised 56 of 993 (5.6%) examinations and were considered potentially management-altering. Discrepancy rates varied significantly by imaging speciality (chi-square (χ²) test, p < 0.001): body imaging (21.6%), musculoskeletal (MSK) imaging (17.2%), neuro-radiological imaging (11.7%), and vascular imaging (5.4%). No statistically significant association was identified between discrepancy rates and resident training grade (χ² test, p = 0.48) or one-hour reporting time block (χ² test, p = 0.37).

Conclusion

This service evaluation showed that provisional radiology reporting is associated with a meaningful discrepancy burden, paying careful attention to variation across imaging specialities. Notably, no statistically significant association was identified between discrepancy rates and resident seniority or time of reporting. Timely consultant review, structured feedback and targeted quality improvement with re-audit will support safe and accurate OOH reporting.

## Introduction

Provisional radiology reports play an important role in guiding the management of acutely unwell patients overnight in hospitals where on-site radiology residents provide acute out-of-hours (OOH) imaging cover. As such, accurate and timely reporting is essential [1-5]. In this context, a reporting discrepancy refers to a difference between the provisional report issued by a radiology resident and the final report provided by the reviewing consultant radiologist [1,4,6]. These discrepancies may range from minor variations with no impact on patient care to significant or major discrepancies that can alter clinical management or delay time-critical interventions [6]. Consequently, reporting discrepancies has the potential to affect clinical management, patient safety and healthcare delivery [3,6,7].

In this evaluation, “discrepancy” refers to a consultant‑perceived discrepancy: any difference identified by the duty consultant radiologist during next‑day review of the on‑call registrar’s provisional overnight report.

At present, there are no defined Royal College, national, or regional standards for acceptable radiology discrepancy rates (DRs). However, an estimated day-to-day reporting error rate of approximately 3% to 5% has been suggested in the literature [1-5]. Published studies report DRs between radiology residents’ provisional CT reports and final consultant reports ranging from 2.0% to 15.1% [1-5]. In the OOH setting, DRs have been reported to be as high as 30% [1]. While some degree of discrepancy in radiological reporting is inevitable, these can be reduced through appropriate training, supervision, and structured quality assurance processes [6]. Importantly, reporting discrepancies may contribute to increased healthcare costs, patient anxiety, and potential patient harm as a result of delayed or inappropriate clinical management [6,7].

Compared with prior work [1-4], this service evaluation focuses on overnight reporting and examines DRs by resident grade, study type, and reporting time to identify areas where targeted interventions may improve diagnostic accuracy in OOH settings.

The aims of this study were as follows: 1) To determine the DRs for overnight on-call reporting at this trust; 2) To evaluate the relationship between DRs and factors such as resident seniority, workload, type of scan, and what time the scan was reported during the night shift.

## Materials and methods

Study design and patient selection

This retrospective study was registered and undertaken as a service evaluation with the service evaluation department at Nottingham University Hospitals NHS Trust, Nottingham, United Kingdom. All consecutive emergency on-call CT and MRI examinations performed at Nottingham University Hospitals NHS Trust, a university teaching hospital and level 1 major trauma centre with over 1,300 beds between 13 June and 3 July 2022 were reviewed.

The inclusion criteria were emergency OOH CT and MRI examinations that were provisionally reported by an on-call radiology resident and subsequently reviewed by a consultant radiologist the following morning. Examinations were excluded if they were reported overnight by a consultant radiologist in the first instance (n=11) or if they were independently reported overnight by a senior on-call resident without subsequent consultant review (n=21). Therefore, a total of 993 imaging reports were analysed (Figure 1).

**Figure 1:**
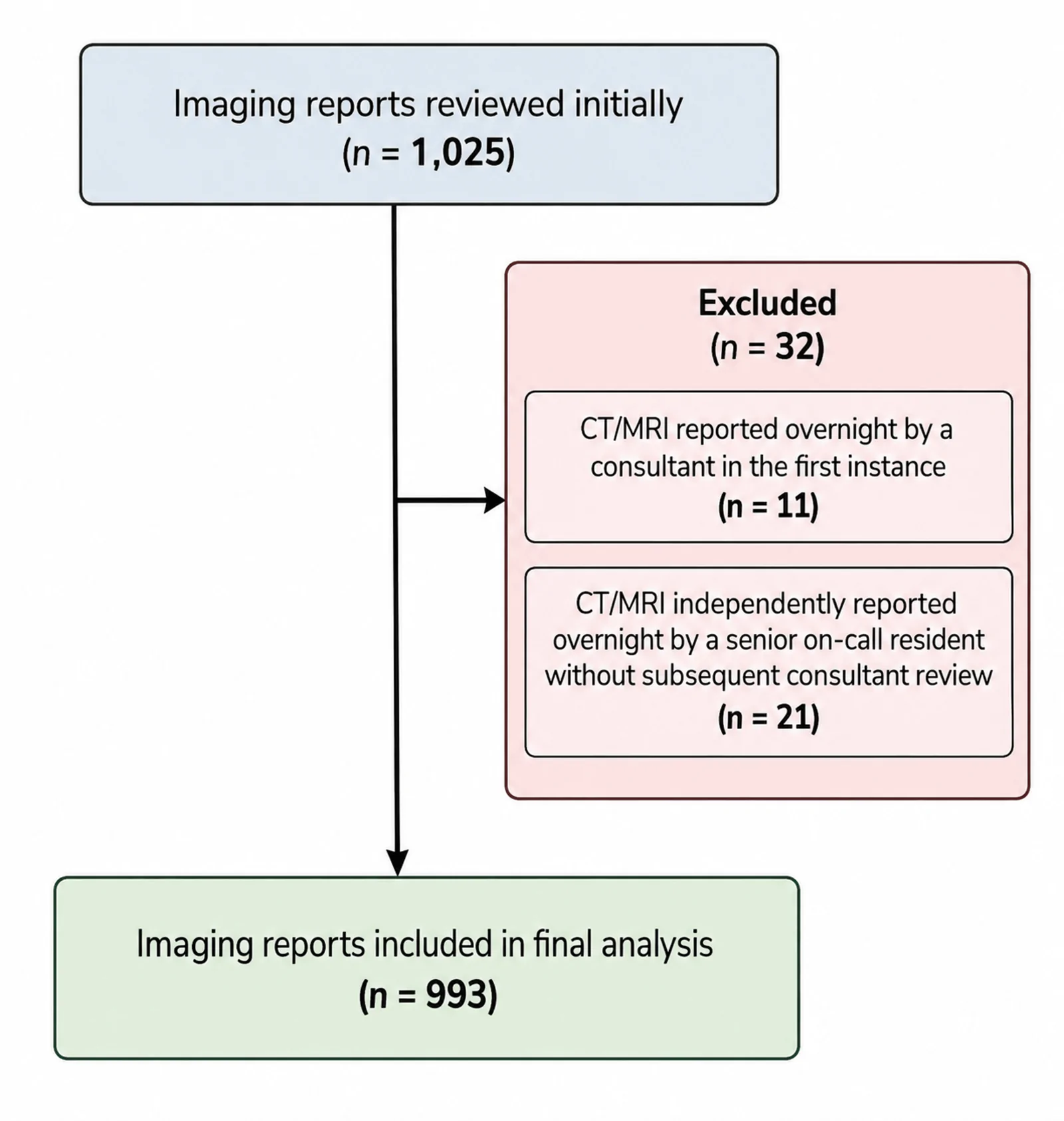
Flow diagram illustrating the selection of imaging examinations included in the study.

Data collection and review of radiology reports

Data were collected retrospectively from the local radiology information system (RIS), picture archiving and communication system (PACS), and electronic patient records, comprising all OOH overnight reports made by on-call radiology residents. Data were collected by a speciality trainee year 4 (ST4) radiology resident, a foundation programme doctor, and two medical students.

Radiology residents reporting these scans included in this study were selected by virtue of being rostered to cover the night on-call shift during the time frame defined previously.

All acute CT and MRI scans provisionally reported overnight were reviewed by a consultant radiologist the following morning. Consultant amendments ranged from minor re-wording to identification of additional findings. Management-altering addenda were communicated to the treating clinical teams using the Trust’s imaging alert process in accordance with local policy.

Forty-eight consultant radiologists reviewed the provisional reports described in this study.

Definitions

DR was calculated as the number of examinations with any consultant-identified discrepancy divided by the total number of examinations reviewed (n=993), multiplied by 100: $\mathrm{DR} = \frac{N_{\mathrm{discrepant}}}{N_{\mathrm{total}}} \times 100$.

For stratified analyses (time block, study type, and resident grade), DRs were calculated within each stratum using the number of examinations in that stratum as the denominator. The formula is as follows: $\mathrm{DR}_{\mathrm{stratum}} = \frac{N_{\mathrm{discrepant,stratum}}}{N_{\mathrm{total,stratum}}} \times 100$.

Discrepancies were classified as minor, significant, or major, defined as follows: (a) Minor, noteworthy findings that would not alter acute management; (b) Significant, findings that could have influenced acute management but were unlikely to result in lasting harm; (c) Major, missed critical findings that would change the course of management and may impact the patient’s long-term outcome.

Discrepancies were broken down further for the major/significant categories into 'missed' or 'misinterpretation'; (a) Miss, defined as a major/significant finding that would alter clinical management but was not mentioned in the original provisional report; (b) Misinterpretation, defined as a finding that was mentioned but not accurately characterised or evaluated

Discrepancy classification was undertaken by an ST4 radiology resident, separate from the reporting cohort included in this study, using the aforementioned predefined criteria.

DRs were also organised into one-hour blocks. The decision to organise the data into one-hour blocks was made to allow a granular assessment of how DR fluctuates throughout the OOH shift and to identify potential temporal trends, such as peaks associated with workload or fatigue.

Statistical analysis

Data were collected and analysed using Microsoft Excel version 16.113 (Microsoft Corporation, Redmond, WA). DRs were calculated and analysed for each resident seniority, each shift segment (split into hours), and each study type. The chi-square (χ²) test was utilised to determine significance.

Ninety-five per cent confidence intervals (CIs) were calculated for the overall DR, the combined major/significant DR and the major DR. Associations between discrepancy status (discrepancy versus no discrepancy) and reporting time, training grade and imaging category were assessed using Pearson’s χ² tests. The time analysis used a 13×2 contingency table (χ²=13.00, df=12, p=0.369), the training-grade analysis a 4×2 table (χ²=2.50, df=3, p=0.476), and the imaging-category analysis a 4×2 table (χ²=21.49, df=3, p<0.001). 

Ethics statement

This project was registered with our trust's service evaluation department. As a service evaluation using anonymised retrospective data, formal research ethics committee approval and individual patient consent were not required in accordance with local policy.

## Results

Study characteristics

A total of 993 imaging examinations were reviewed, with 144 consultant‑perceived discrepancies identified (overall DR 14.5%). The included examinations comprised acute CT and MRI scans. The examinations were organised into the following subspecialist types to reflect the various radiology specialist interests: Musculoskeletal (MSK) radiology, comprising MR spine, trauma CT (MSK trauma), and preoperative surgical planning CT; Neuroradiology, comprising CT head (including CT head examinations from trauma CT) and CT intracranial angiography; Body radiology, comprising CT thorax, abdomen and pelvis (TAP) (including CT TAP examinations from trauma CT), CT pulmonary angiography (CTPA), and CT kidneys, ureters and bladder (KUB); and Vascular radiology, comprising CT aorta, CT mesenteric angiography, and CT peripheral angiography.

Discrepancy rates

The overall DR was found to be 144 (14.5%), consisting of 18 (1.8%) major discrepancies, 38 (3.8%) significant discrepancies, and 88 (8.9%) minor discrepancies (Table 1). Management-altering DR (major/significant combined) was 56 (5.6%). The mean number of discrepancies per overnight shift was 6.9 (144 discrepancies across 21 shifts).

**Table 1:** Summary of discrepancy rates (DRs)

	Major	Significant	Minor	Total
N	18	38	88	144
DR (N (%))	18 (1.8%)	38 (3.8%)	88 (8.9%)	144 (14.5%)
Proportion of all discrepancies (N (%))	18 (12.5%)	38 (26.4%)	88 (61.1%)	144 (100%)

Associations with DRs

DR vs Time

DRs were analysed in one‑hour blocks (Table 2). The relationship between reporting volume and the number of discrepancies identified across the overnight period is illustrated in Figure 2. Reporting volume and DR varied across the shift; however, differences between time blocks were not statistically significant (χ² test, p=0.37).

**Table 2:** Discrepancy rates organised into one-hour blocks

Time block	Major n (%)	Significant n (%)	Minor n (%)	Total n (%)	Scans (n)
8 pm–9 pm	0 (0.0%)	0 (0.0%)	0 (0.0%)	0 (0.0%)	18
9 pm–10 pm	3 (3.3%)	3 (3.3%)	8 (8.9%)	14 (15.6%)	90
10 pm–11 pm	1 (0.9%)	5 (4.5%)	11 (9.8%)	17 (15.2%)	112
11 pm–12 am	0 (0.0%)	6 (4.4%)	14 (10.4%)	20 (14.8%)	135
12 am–1 am	4 (3.7%)	4 (3.7%)	7 (6.5%)	15 (14.0%)	107
1 am–2 am	3 (3.0%)	4 (4.0%)	10 (10.1%)	17 (17.2%)	99
2 am–3 am	1 (1.1%)	6 (6.5%)	10 (10.9%)	17 (18.5%)	92
3 am–4 am	1 (1.3%)	1 (1.3%)	4 (5.1%)	6 (7.6%)	79
4 am–5 am	0 (0.0%)	1 (1.4%)	6 (8.3%)	7 (9.7%)	72
5 am–6 am	1 (1.7%)	1 (1.7%)	9 (15.0%)	11 (18.3%)	60
6 am–7 am	2 (3.6%)	3 (5.4%)	5 (8.9%)	10 (17.9%)	56
7 am–8 am	1 (1.9%)	2 (3.8%)	2 (3.8%)	5 (9.6%)	52
8 am–9 am	1 (4.8%)	2 (9.5%)	2 (9.5%)	5 (23.8%)	21
Total	18 (1.8%)	38 (3.8%)	88 (8.9%)	144 (14.5%)	993

**Figure 2:**
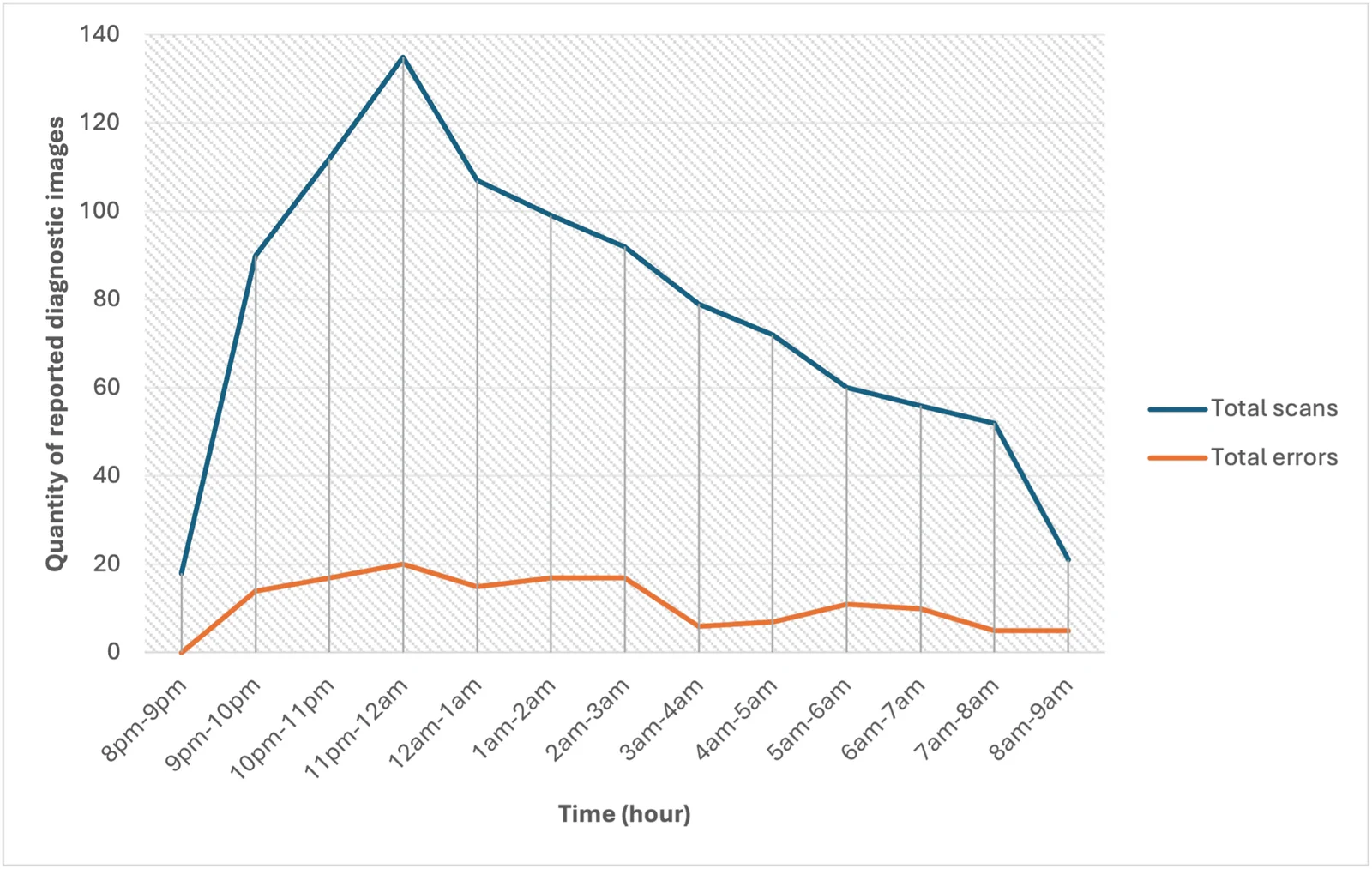
Discrepancy rates vs total number of scans overnight (by hour)

DR vs Study Type

Speciality category: Body radiology demonstrated the highest within-speciality DR (60 (21.6%) of 278 examinations), followed by MSK radiology (20 (17.2%) of 116 examinations), neuroradiology (59 (11.7%) of 506 examinations), and vascular radiology (5 (5.4%) of 93 examinations). Although neuroradiology had a lower within-speciality DR than body radiology, it accounted for a large absolute discrepancy burden due to volume (59 (41.0%) of 144 total discrepancies), similar to body radiology (60 (41.7%) of 144 total discrepancies) (Figure 3). Differences in overall DR across specialities were statistically significant (χ² test, p<0.001).

**Figure 3:**
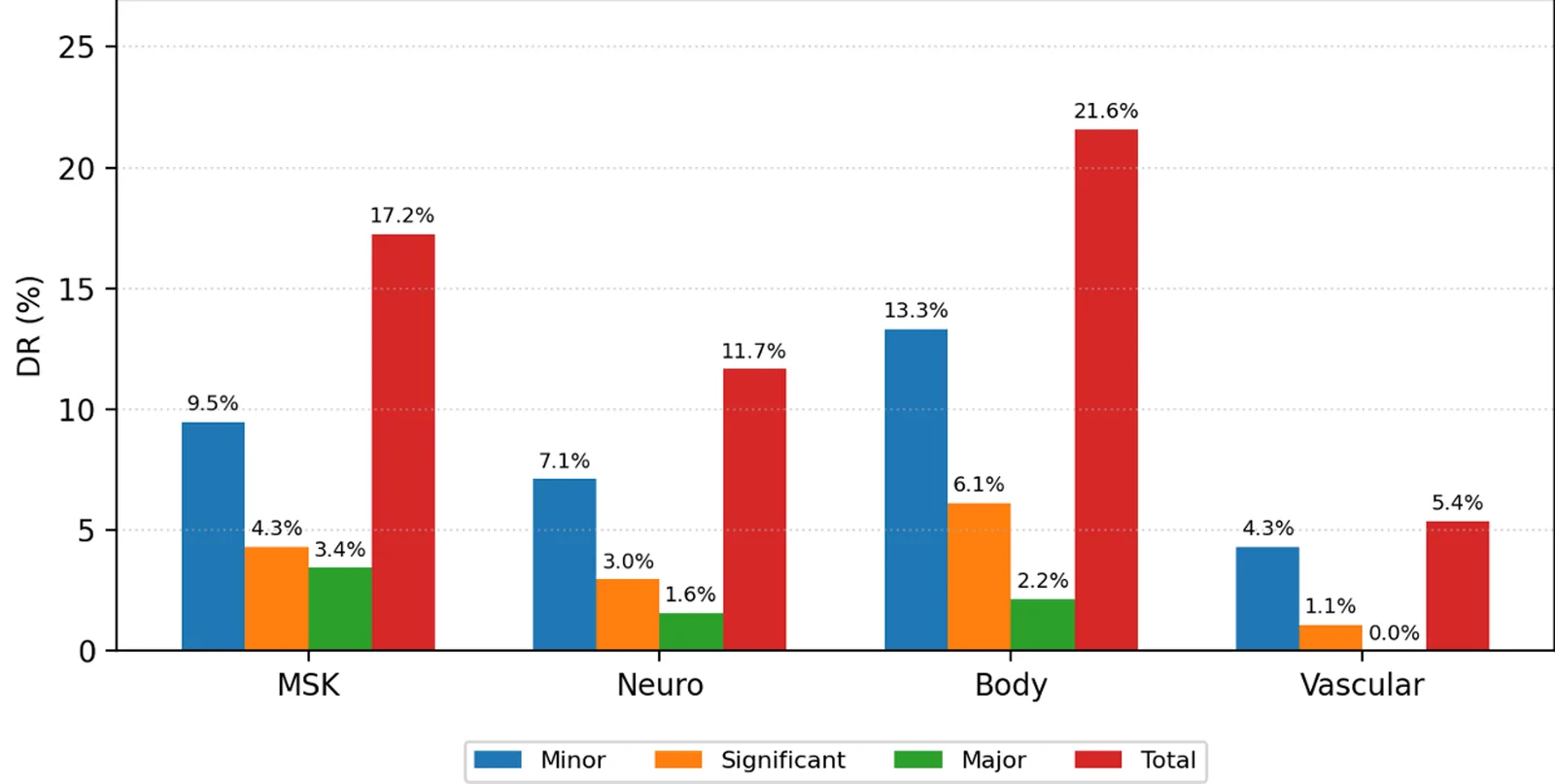
Discrepancy rates (DR) vs study type (within category) MSK: musculoskeletal

Within each category, total DRs were highest for body (21.6%) and MSK (17.2%) examinations, and lowest for vascular examinations (5.4%) (Table 3). Body examinations contributed the largest absolute number of discrepancies, while neuro-radiological scan reporting showed most major discrepancies. No major discrepancies were recorded in vascular examinations during the study period.

**Table 3:** Discrepancy rates (DR) for each body part

Speciality category	Major discrepancy n (% of all scans)	Significant discrepancy n (% of all scans)	Minor discrepancy n (% of all scans)	Total discrepancies n (% of all scans)	Total scans n (% of all scans)	Within-speciality DR (%)	Share of all discrepancies n (%)
Musculoskeletal (MSK) radiology	4 (0.4%)	5 (0.5%)	11 (1.1%)	20 (2.0%)	116 (11.7%)	17.2%	20 (13.9%)
Neuroradiology (Neuro)	8 (0.8%)	15 (1.5%)	36 (3.6%)	59 (5.9%)	506 (51.0%)	11.7%	59 (41.0%)
Body radiology (Body)	6 (0.6%)	17 (1.7%)	37 (3.7%)	60 (6.0%)	278 (28.0%)	21.6%	60 (41.7%)
Vascular radiology (Vascular)	0 (0.0%)	1 (0.1%)	4 (0.4%)	5 (0.5%)	93 (9.4%)	5.4%	5 (3.5%)

DR vs Resident Seniority

Eleven residents reported scans over the 21 overnight shifts analysed: ST2 (4, 36.4%), ST3 (2, 18.2%), ST4 (2, 18.2%), and ST5 (3, 27.3%), with percentages representing the proportion of residents in each training grade. Across the study period, ST5 residents issued the largest volume of provisional reports (430, 43.3%).

When DRs were calculated within each training grade, the total DR ranged from 44 (12.7% of 346 scans reported by ST2s) to 17 (17.9% of 95 scans reported by ST3s) and 21 (17.2% of 122 scans reported by ST4s), with ST5 at 62 (14.4% of 430 scans reported by ST5s) (Table 4). The major DR within each training grade was 4 (1.2%) for ST2, 1 (1.1%) for ST3, 5 (4.1%) for ST4 and 8 (1.9%) for ST5. Notably, no statistically significant association was identified between DRs and resident training grade (χ² test, p = 0.48)

**Table 4:** Discrepancy rates (DRs) vs resident seniority ST: specialty trainee

Year of training	Major discrepancy n (% of all scans)	Significant discrepancy n (% of all scans)	Minor discrepancy n (% of all scans)	Total discrepancies n (% of all scans)	Total scans n (% of all scans)	Within‑grade DR (%)	Share of all discrepancies n (%)
ST2	4 (0.4%)	12 (1.2%)	28 (2.8%)	44 (4.4%)	346 (34.8%)	12.7%	44 (30.6%)
ST3	1 (0.1%)	4 (0.4%)	12 (1.2%)	17 (1.7%)	95 (9.6%)	17.9%	17 (11.8%)
ST4	5 (0.5%)	7 (0.7%)	9 (0.9%)	21 (2.1%)	122 (12.3%)	17.2%	21 (14.6%)
ST5	8 (0.8%)	15 (1.5%)	39 (3.9%)	62 (6.2%)	430 (43.3%)	14.4%	62 (43.1%)

Given the limited sample size within each grade and the likelihood of case-mix differences, these comparisons should be interpreted cautiously. However, the combination of higher reporting volume and sustained DRs supports further exploration of workload, interruptions, and case complexity as contributors to overnight discrepancies.

Type of discrepancy among major and significant reporting discrepancies

As displayed in Table 5, of the 56 major and significant discrepancies, 45 (80.4%) were classified as missed findings and 11 (19.6%) as misinterpretations. Missed findings accounted for 15 (83.3%) of 18 major discrepancies and 30 (78.9%) of 38 significant discrepancies. The distribution of discrepancy types was similar between severity categories. No statistically significant association was identified (p=1.00).

**Table 5:** Type of discrepancy among major and significant reporting discrepancies

Discrepancy severity	Miss, n (%)	Misinterpretation, n (%)	Total, n
Major	15 (83.3%)	3 (16.7%)	18
Significant	30 (78.9%)	8 (21.1%)	38
Total	45 (80.4%)	11 (19.6%)	56

A note on patient outcomes at the two-year follow-up

A two-year outcomes follow-up was carried out by reviewing follow-up imaging and the clinical notes for the discrepant cases identified from this service evaluation. This was conducted by accessing patient notes on the electronic patient record. The review did not identify documented patient harm, including those cases that were deemed to have a major discrepancy within the provisional report. For all these cases, the consultant review the following morning ensured timely correction and appropriate patient management as necessary.

## Discussion

Why monitor discrepancies?

In this three-week service evaluation of 993 overnight imaging examinations, the overall DR between registrar provisional reports and consultant final reports was 144 scans (14.5% of 993). Significant discrepancies comprised 56 (5.6%) examinations and were considered management-altering. DRs did not differ significantly across one-hour time blocks (χ² test, p=0.37) or by training grade (χ² test, p=0.48).

The overall DR of 14.5% is comparable with rates reported in previous studies of OOH and provisional radiology reporting, although substantial variation has been described between institutions and imaging populations [1-5]. Importantly, 5.6% of examinations contained major or significant discrepancies, demonstrating that a relatively small proportion of discrepant reports may still have potential clinical relevance in an emergency reporting environment. Our findings support previous literature highlighting discrepancy monitoring as an important component of quality assurance and trainee education, while also demonstrating the value of considering the severity of perceived discrepancies alongside overall DRs when assessing the clinical significance of overnight reporting [1,3,6,7].

Analysing DRs within a department is important for evaluating reporting performance, identifying training needs and mitigating risks to patient safety. This is particularly relevant in high-volume emergency and trauma settings, where overnight CT reporting supports time-critical decision-making and rapid clinical triage.

There is currently no universally agreed UK benchmark for DRs in overnight provisional radiology reporting. Previous studies have reported substantial variation in DRs, with DRs ranging between 2.0-15.1% [1-5]. This reflects the influence of factors such as radiologist experience, case complexity, workload, and reporting environment. Cognitive factors may also contribute to discrepancies, as evidenced by the literature previously [8]. The variability in DRs, as well as their causation, also highlights the importance of interpreting DRs in the context of local service configuration, rather than applying a single benchmark across different institutions.

DRs by speciality

Neuro-radiological imaging examinations constituted the largest proportion of the overnight workload (506, 51% of 993 total scans reviewed). Although the within-category DR for neuro-radiological examinations of 59 (11.7%) was marginally lower than that observed for body (60, 21.6%) and MSK examinations (20, 17.2%), the high volume of head CT examinations meant that neuro-radiological imaging contributed the greatest absolute number of major discrepancies (8, 44.4%). This is consistent with prior studies demonstrating higher DRs in emergency head CT interpretation by residents compared with consultant radiologists [9-13].

DRs by training grade

Within grade DRs varied modestly by training grade (ST2 12.7%, ST3 17.9%, ST4 17.2%, ST5 14.4%); however, this association was not statistically significant (χ² test, p=0.48).

ST5 registrars contributed both the greatest share of total discrepancies (62, 43.1% of a total of 144) and reflected the largest share of overnight reporting volume (430 (43.3%) of a total of 993 examinations).

Several factors may contribute to the modest variation observed by training grade. More senior registrars may undertake a higher proportion of complex examinations and may be more likely to issue definitive provisional overnight reports rather than defer interpretation, increasing the opportunity for discrepancies to be identified at next day consultant review. Future work should adjust for case mix (including modality and clinical complexity) to better characterise any relationship between seniority, workload and discrepancy severity.

DRs by time of shift

Although DRs differed numerically across time blocks, these differences were not statistically significant (χ² test, p=0.37), and there was no consistent 'late shift increase' suggestive of fatigue alone, contrasting with comparable studies [14].

This suggests that time on task alone may not explain discrepancy patterns in this setting. Workload and interruptions may also contribute [15], although these factors were not directly measured in the present study.

The descriptive peak in discrepancy numbers during the middle of the night (11 pm-12 am) reflects the busiest period of scans overall; at this time, the number of scans reported overnight (n=135) is the largest proportion of scans reported in an hour across the three-week time period. This higher workload is also when on-call registrars balance reporting with protocoling, urgent clinical communication and coordination tasks. 

Fatigue has been identified as a potential contributor to radiological reporting error [14,16]. Visual strain is also recognised during prolonged image interpretation and may affect the reporting environment [17]. While this evaluation did not demonstrate a consistent 'late-shift' effect, practical measures such as optimising workstation ergonomics, promoting screen breaks, and minimising avoidable interruptions may still help support accuracy during overnight work [18].

Impact on patient outcomes

Overall, 5.6% of the 993 cases reviewed in this service evaluation may have resulted in altered inpatient management had scans been reported initially by a consultant; however, only 1.8% were associated with a perceived 'major' discrepancy with the potential to delay or alter management. Reassuringly, two-year follow-up of discrepant cases identified no documented patient harm. This may reflect timely consultant review and effective escalation pathways, although causality cannot be established from this retrospective evaluation. Despite this, reducing the frequency of discrepancies that may alter management remains an important patient safety priority.

Limitations

Firstly, discrepancies were identified during routine next-day duty review and were not adjudicated through a formal discrepancy panel. As such, the findings represent consultant-perceived discrepancies and may be influenced by inter-observer variation. Secondly, detailed classification of discrepancies relied on one ST4 registrar. Future studies may wish to minimise bias and improve reliability of classification by introducing a secondary or more senior reviewer for this classification stage.

As previously discussed, although more senior residents demonstrated higher DRs, this study did not assess the complexity of reported cases or determine whether more junior residents selectively reported less complex examinations, potentially introducing selection bias. Nevertheless, it is reasonable to infer that the higher DRs observed among the most senior residents are at least partly attributable to their increased reporting workload.

Finally, this study analysed scans reported over a three-week period in 2022 and included only 11 residents, representing a subset of the 41 residents participating in the on-call rota. As such, the findings may not be fully representative of the entire resident cohort.

## Conclusions

This service evaluation demonstrates that provisional OOH diagnostic imaging reporting by radiology resident doctors does indeed carry a meaningful discrepancy burden, in particular with variation across imaging specialities. However, it is important to note that DRs were not significantly associated with reporting doctor seniority or time of reporting. This substantiates the idea that discrepancies cannot be attributed simply to trainee experience or fatigue. The variation between specialities and contribution of reporting volume highlights the importance of considering the wider reporting environment, including workload and variation in scan type, when interpreting discrepancy patterns.

The absence of documented patient harm over two years is reassuring; however, this does not negate the importance of identifying and learning from discrepancies that have the potential to alter management. Timely consultant review provides a vital safety net, while structured feedback and targeted education can turn discrepancies into opportunities for learning and improvement. Continued local monitoring, quality improvement and re-audit are therefore important not only for measuring DRs but also for identifying patterns and strengthening the systems that support safe and accurate OOH reporting by our radiology resident doctors.
